# Supplementary material for: Biocatalytic nanoparticles for the stabilization of degassed single electron transfer-living radical pickering emulsion polymerizations
Source: Nat Commun. 2020 Nov 5;11:5599. doi: 10.1038/s41467-020-19407-3 (PMC7645627; doi:10.1038/s41467-020-19407-3)
Supplement: Supplementary file 1 — Supplementary Information [file 41467_2020_19407_MOESM1_ESM.pdf]

Supplementary Information

Biocatalytic Nanoparticles for the Stabilization of Degassed Single Electron Transfer Living Radical Pickering Emulsion Polymerizations

Adrian Moreno*[1] and Mika H. Sipponen*[1]

[1] Department of Materials and Environmental Chemistry, Stockholm University, SE-10691, Stockholm, Sweden.

*Corresponding authors:

adrian.morenoguerra@mmk.su.se

mika.sipponen@mmk.su.se

This PDF file includes:

Supplementary Figures.. 2
Supplementary Tables.. 12
Supplementary Methods.. 15
Supplementary References.. 17

Supplementary figures

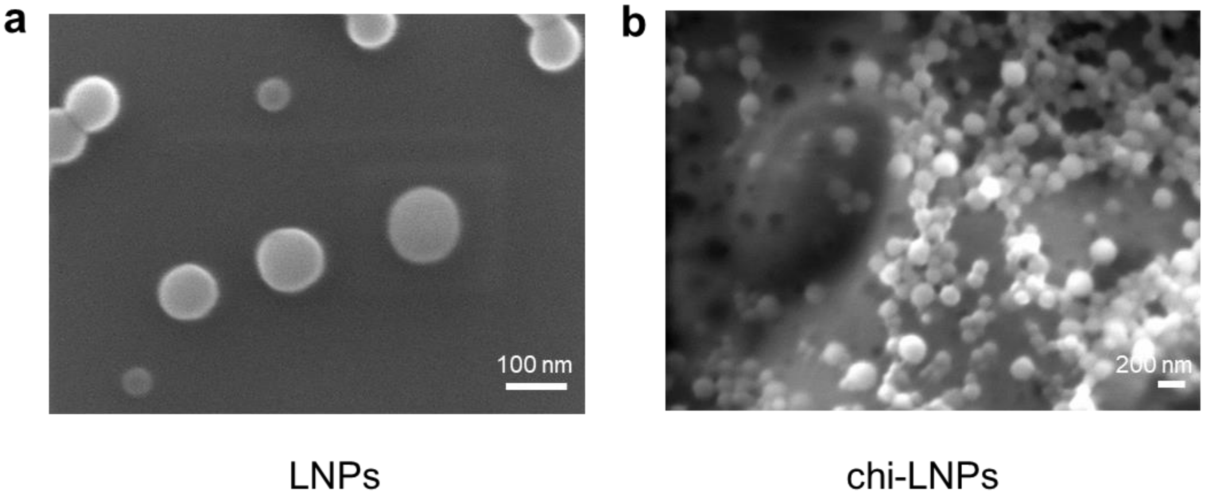

Supplementary Figure 1. SEM images: (a) LNPs and (b) chi-LNPs.

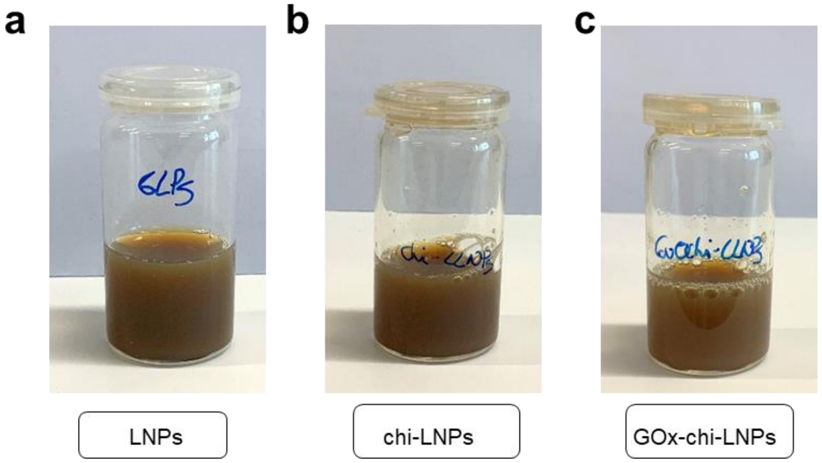

Supplementary Figure 2. Appearance of colloidal dispersions of lignin particles used in this work: (a) LNPs, (b) chi-LNPs, (c) GOx-chi-LNPs.

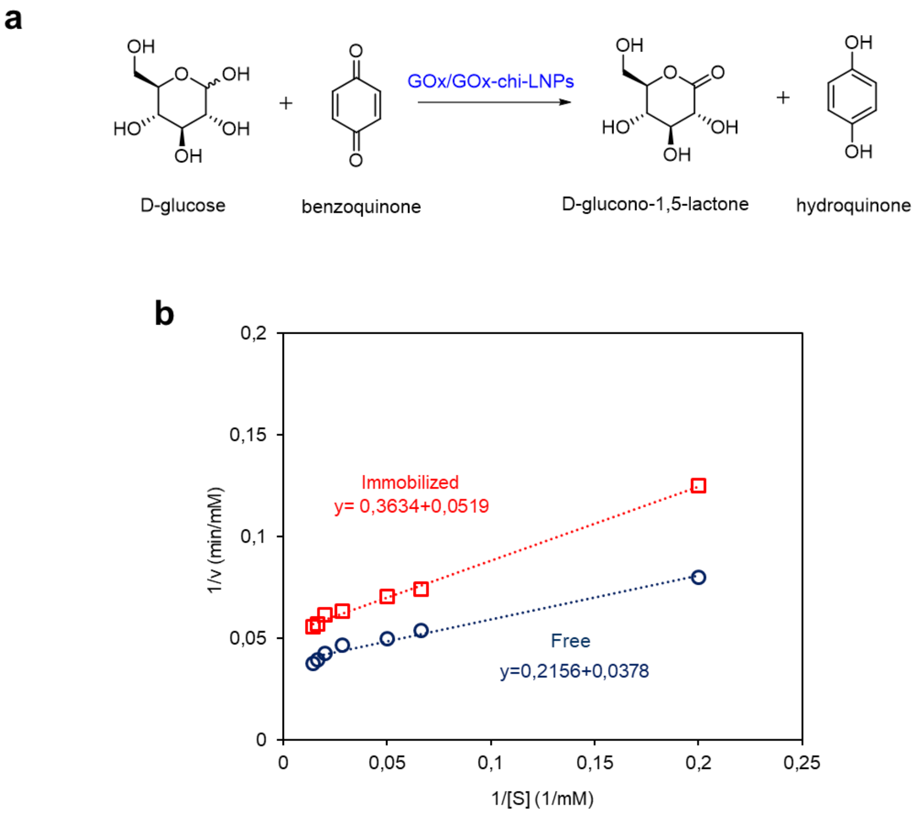

Supplementary Figure 3. Enzyme activity assay of GOx: (a) Scheme of enzymatic reaction used to determine the enzyme activity on GOx-chi-LNPs. (b) The Lineweaver-Burk plots for the (circles) free GOx enzyme and (squares) enzyme-coated LNPs (GOx-chi-LNPs) measured at 25 ºC.

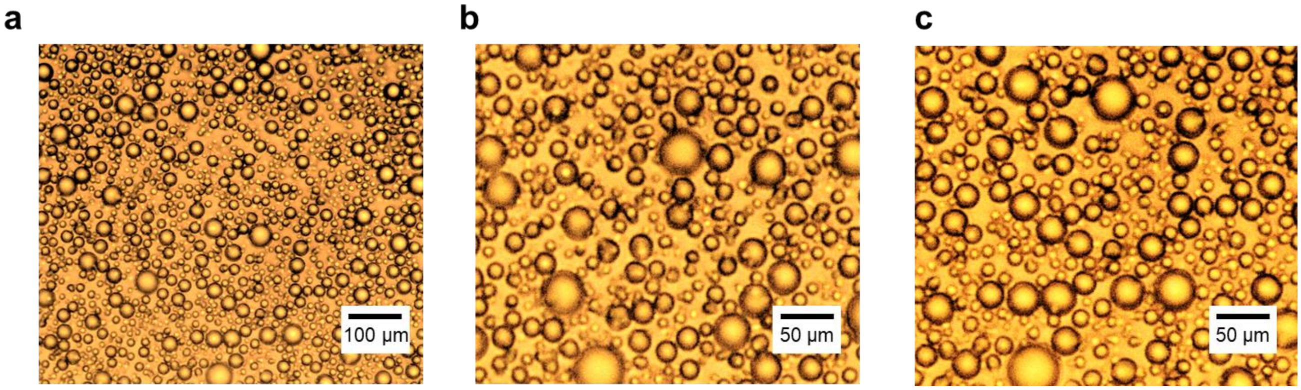

Supplementary Figure 4. Analysis of Pickering emulsions by optical microscope: (a) butyl methacrylate (BMA), (b) styrene (S) and (c) methyl acrylate (MA)-Pickering emulsions stabilized by GOx-chi-LNPs at 20 g of GOx-chi-LNPs per L of monomer.

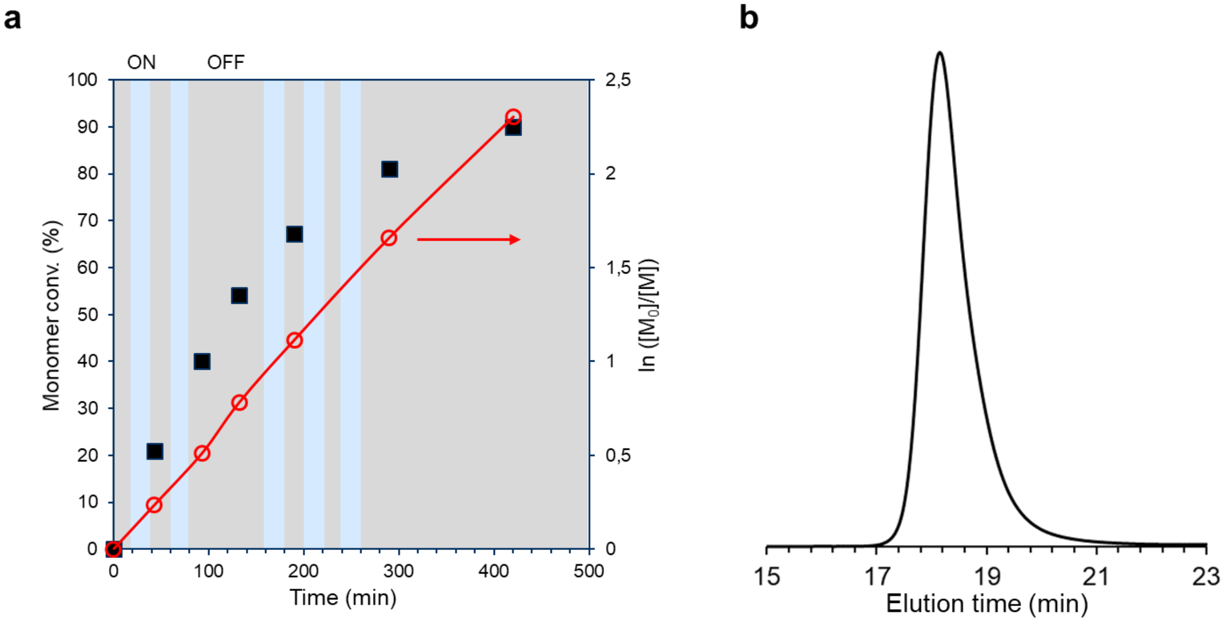

Supplementary Figure 5. Temporal control in on-off study for an open-closed vial system: (a) Kinetic plot for the enzymatic-degassed SET-LRP-mediated Pickering emulsion of BMA in temporal control of on-off study in open-closed vial system. Reaction conditions: $[BMA]_0/[MBPA]_0/[Me_6\text{-}TREN]_0/[Cu(0)]_0$ = 200/1/0.2/0.3. [GOx-chi-LNPs] = 2.25 wt % relative to BMA. (b) GPC trace of the final PBMA after the Pickering emulsion SET-LRP process.

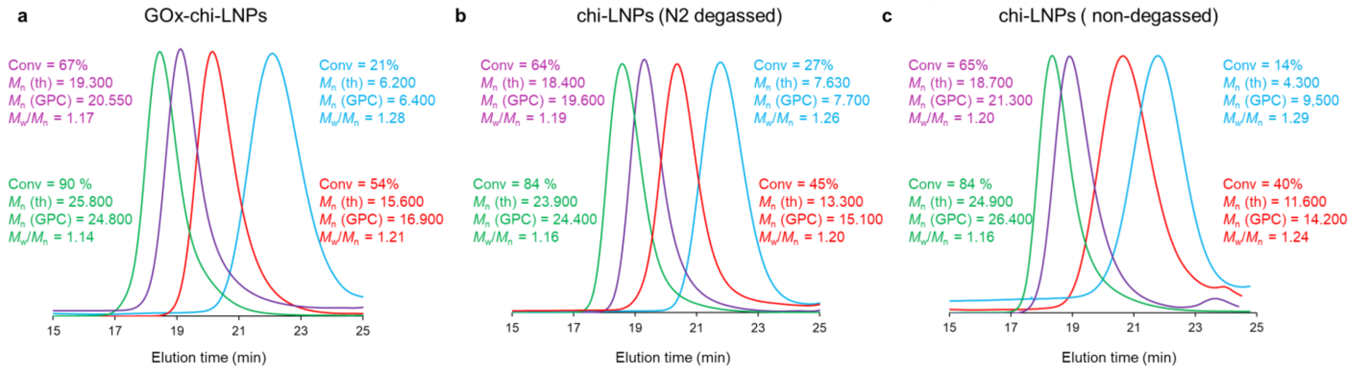

Supplementary Figure 6. GPC analysis of PBMA polymers: GPC traces of PBMA obtained via SET-LRP-mediated Pickering emulsion of BMA catalyzed by Cu(0) powder using lignin particles as emulsifier: (a) GOx-chi-LNPs, (b) chi-LNPs (applying N2 as degassing methodology) and (c) chi-LNPs (no degassing methodology applied).

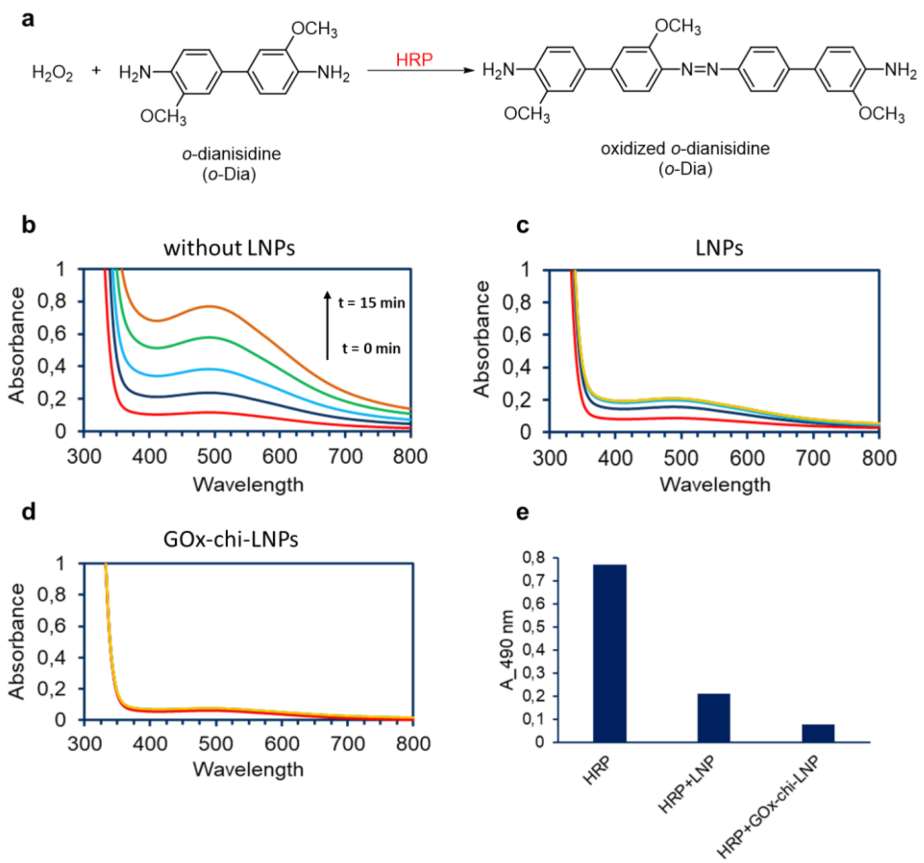

Supplementary Figure 7. H_2O_2 scavenging behavior of LNPs: (a) Scheme of enzymatic model reaction used to determine the ability of GOx-chi-LNPs to consume H_2O_2. UV-visible spectra in control reactions: (a) without the presence of lignin particles, (b) in the presence of LNPs and (c) in the presence of GOx-chi-LNPs. (e) Absorbance of oxidized *o*-dianisidine after 15 min of reaction with and without the presence of lignin particles. Reaction conditions: [H_2O_2] = 20 mM, [*o*-dianisidine] = 9.1 mM, [HRP] = 12.5 μg/mL and [lignin particles] = 30μg/mL. Reaction media: pH 6 in 15 mM NaOAc buffer. Total volume reaction = 3 mL

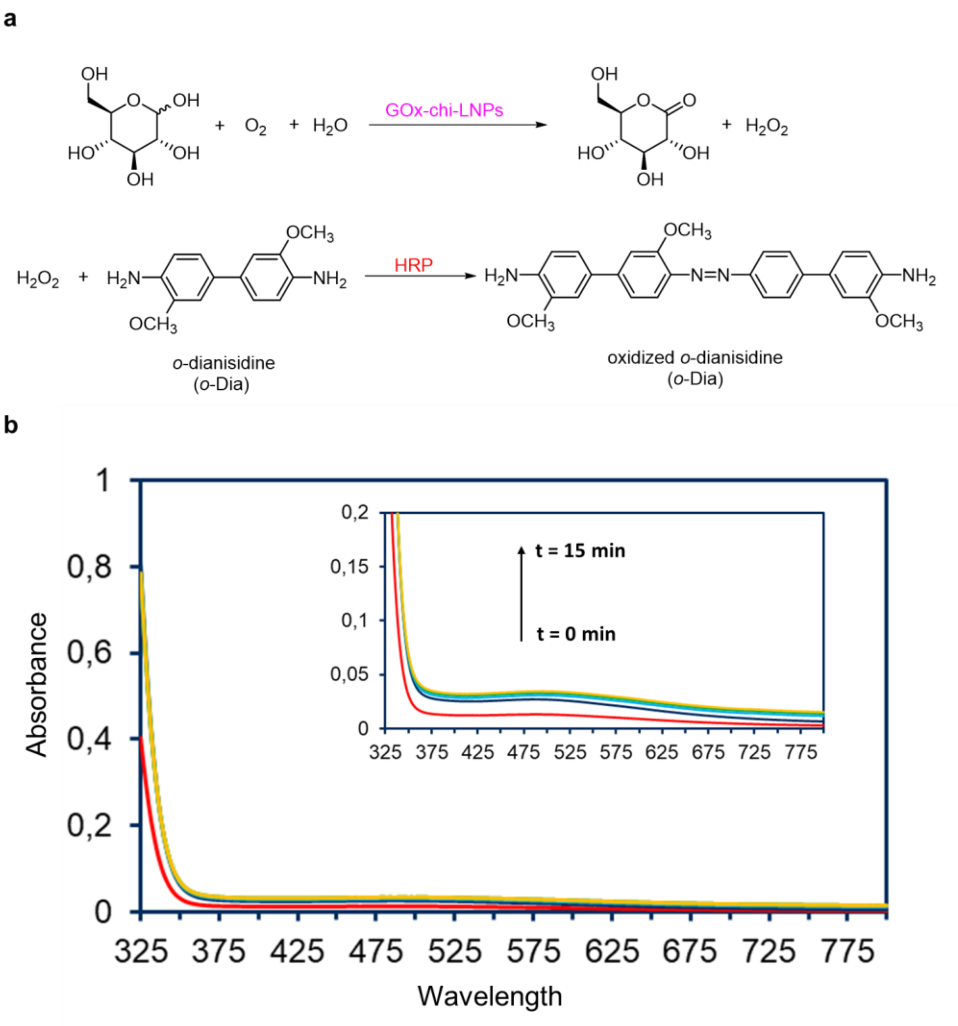

Supplementary Figure 8. H_2O_2 scavenging behavior of GOx-chi-LNPs during the polymerization: (a) Scheme of enzymatic tandem model reaction used to determine the ability of GOx-chi-LNPs to consume *in situ* generated H_2O_2 during degassing step during the polymerization process. (b) UV-visible spectra of reaction mixture recorded at different times. Reaction conditions: [BMA] = 1.78 g (20% vol), [glucose] = 0.14 M, [GOx-chi-LNPs] = 2.5 wt % respect to BMA, [HRP] = 20mg/mL and [*o*-dianisidine] = 9.1 mM Reaction media: pH 6 in 15 mM NaOAc buffer. Total volume reaction = 10 mL.

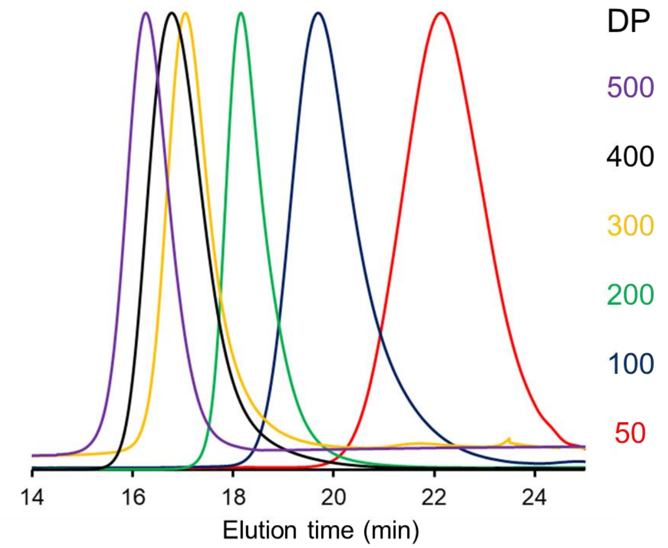

Supplementary Figure 9. GPC traces (normalized to peak height) of PBMA with different targeted DPs (50-500) obtained by enzyme-degassed SET-LRP of BMA in Pickering emulsion phase.

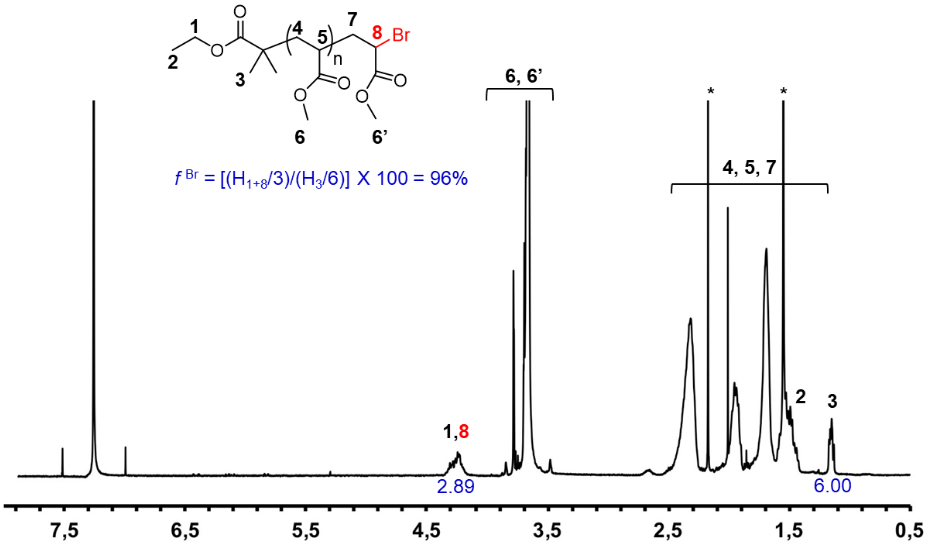

Supplementary Figure 10. ^{1}H NMR spectrum of PMA-Br (M/I = 50) in $CDCl_3$ at 25 °C obtained by SET-LRP-mediated Pickering emulsion using GOx-chi-LNPs as emulsifiers. Reaction conditions $[MA]_0/[EBiB]_0/[Me_6\text{-}TREN]_0/[Cu(0)]_0$ = 50/1/0.2/0.3. [GOx-chi-LNPs] = 2.25 wt % relative to MA. *denotes the signals from residual solvents (water and acetone).

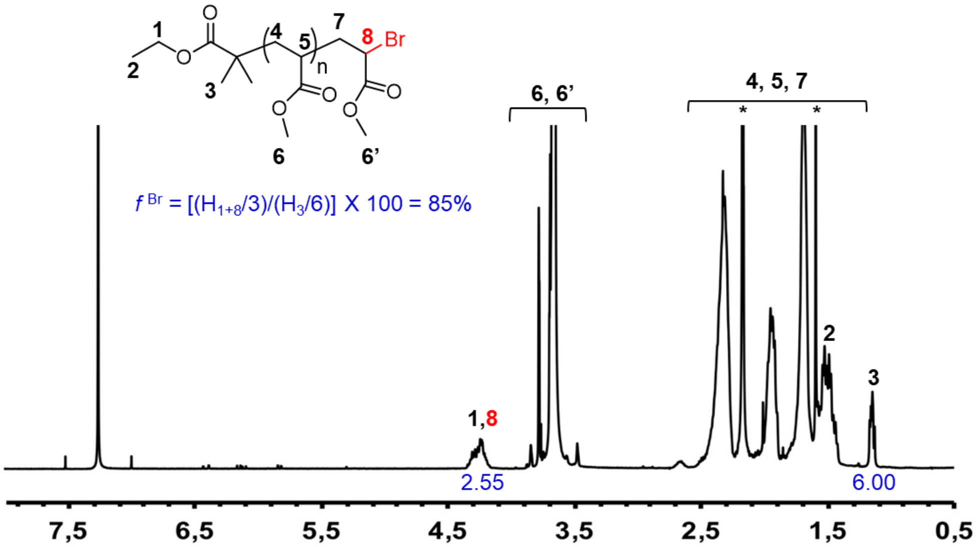

Supplementary Figure 11. ^{1}H NMR spectrum of PMA-Br (M/I = 50) in $CDCl_3$ at 25 °C obtained by SET-LRP-mediated Pickering emulsion using chi-LNPs as emulsifiers in presence of air. Reaction conditions $[MA]_0/[EBiB]_0/[Me_6\text{-}TREN]_0/[Cu(0)]_0$ = 50/1/0.2/0.3. [chi-LNPs] = 2.25 wt % relative to MA. *denotes the signals from residual solvents (water and acetone).

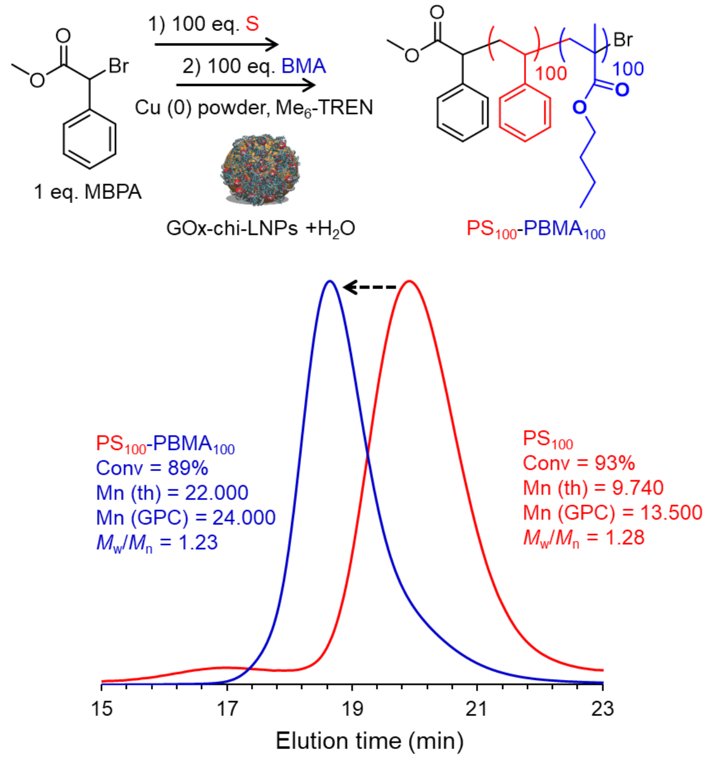

Supplementary Figure 12. GPC analysis of enzyme-degassed SET-LRP-mediated Pickering emulsion block copolymerization *via* purification-reinitiation strategy of S with BMA to synthetize PS-PBMA block copolymer. Reaction conditions for the polystyrene (PS) macroinitiator: $[S]_0/[MBPA]_0/[Me_6\text{-TREN}]_0/[Cu(0)]_0$ = 100/1/0.2/0.3. [GOx-chi-LNPs] = 2.25 wt % relative to S.

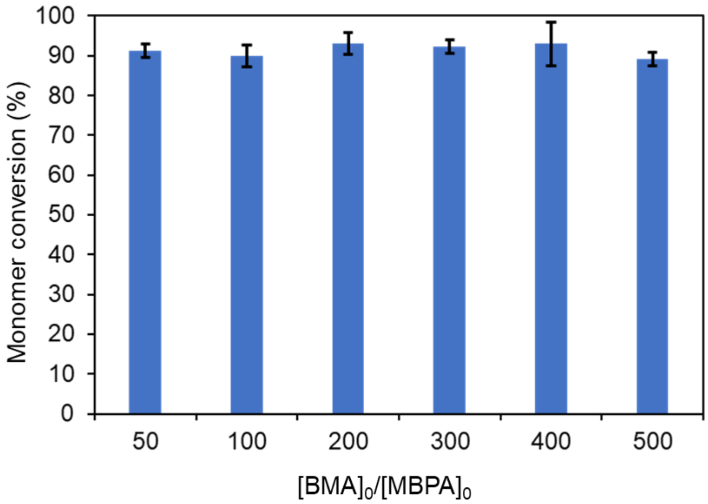

Supplementary Figure 13. Conversion of BMA with different targeted DPs (50-500) obtained by enzyme-degassed SET-LRP-mediated Pickering emulsion of BMA. Reaction time 24 h.

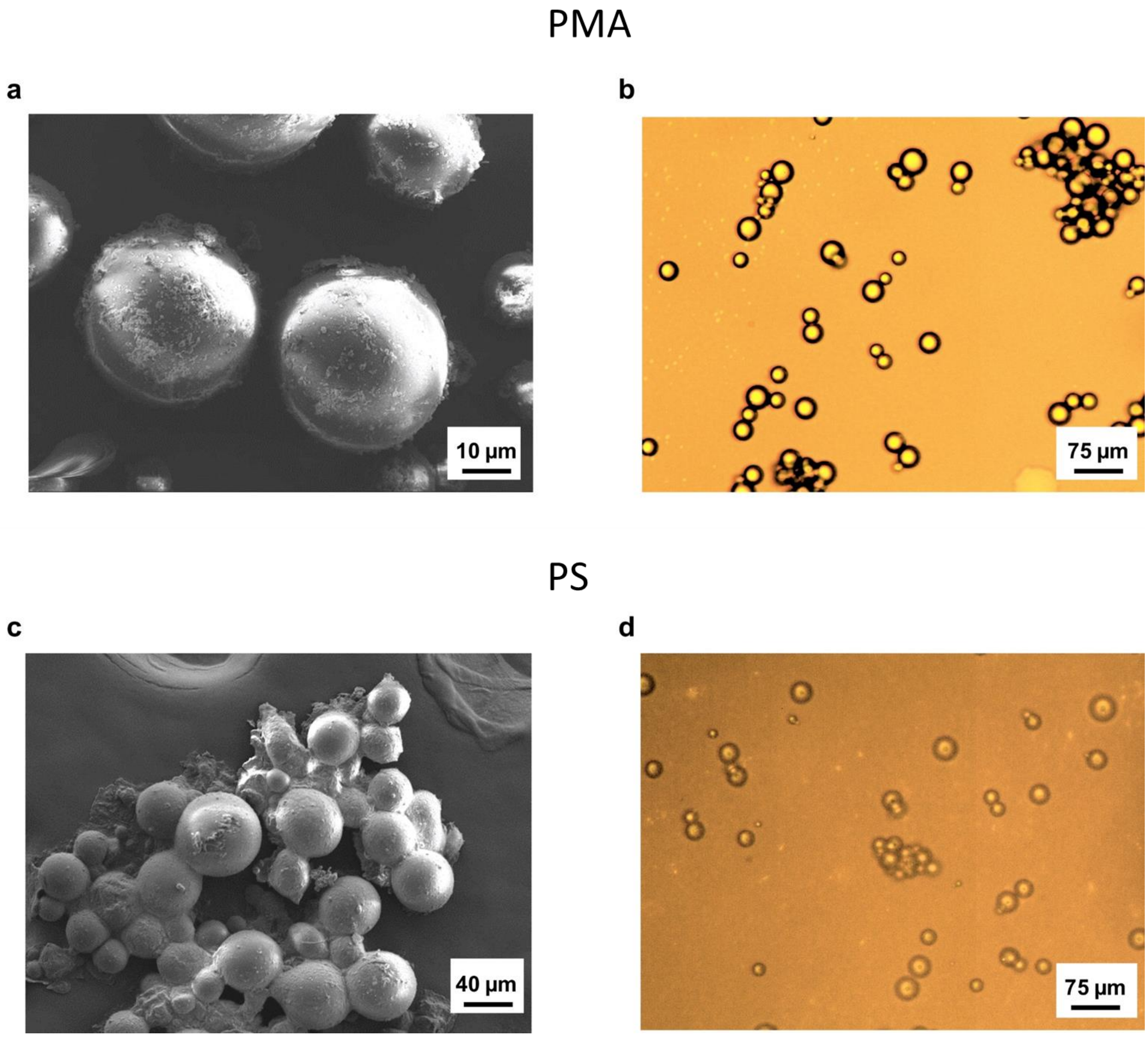

Supplementary Figure 14. SEM images of lignin-coated (a) Poly(methyl acrylate) (PMA) and (c) Polystyrene (PS) microbeads after SET-LRP process. Optical microscopic images of (b) PMA and (d) PS latex dispersions obtained after SET-LRP process.

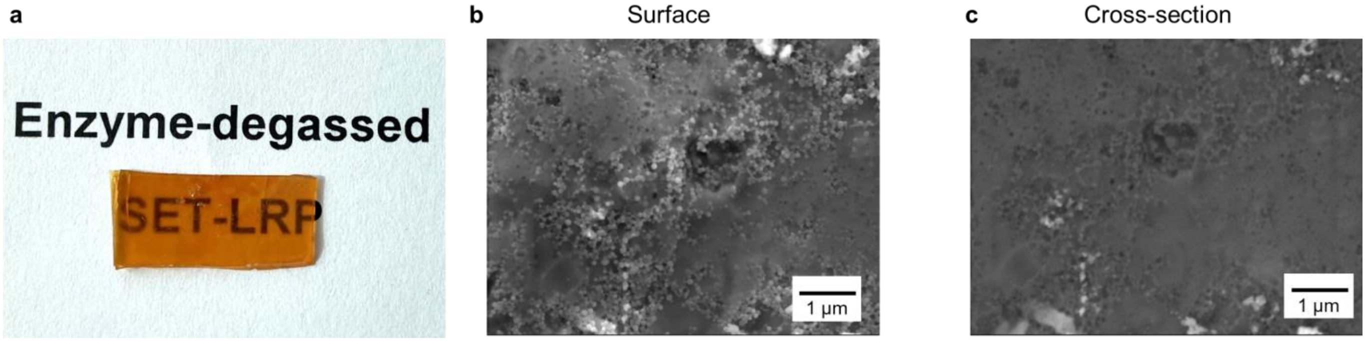

Supplementary Figure 15. (a) Digital image of PBMA-GOx-chi-LNPs composite after melting process (160 ºC) of BMA latex dispersion stabilized with GOx-chi-LNPs (6 wt % relative to BMA). Font size corresponds to Arial, 11 pt. (b and c) SEM images of surface and cross-section of PBMA-GOx-chi-LNPs composite.

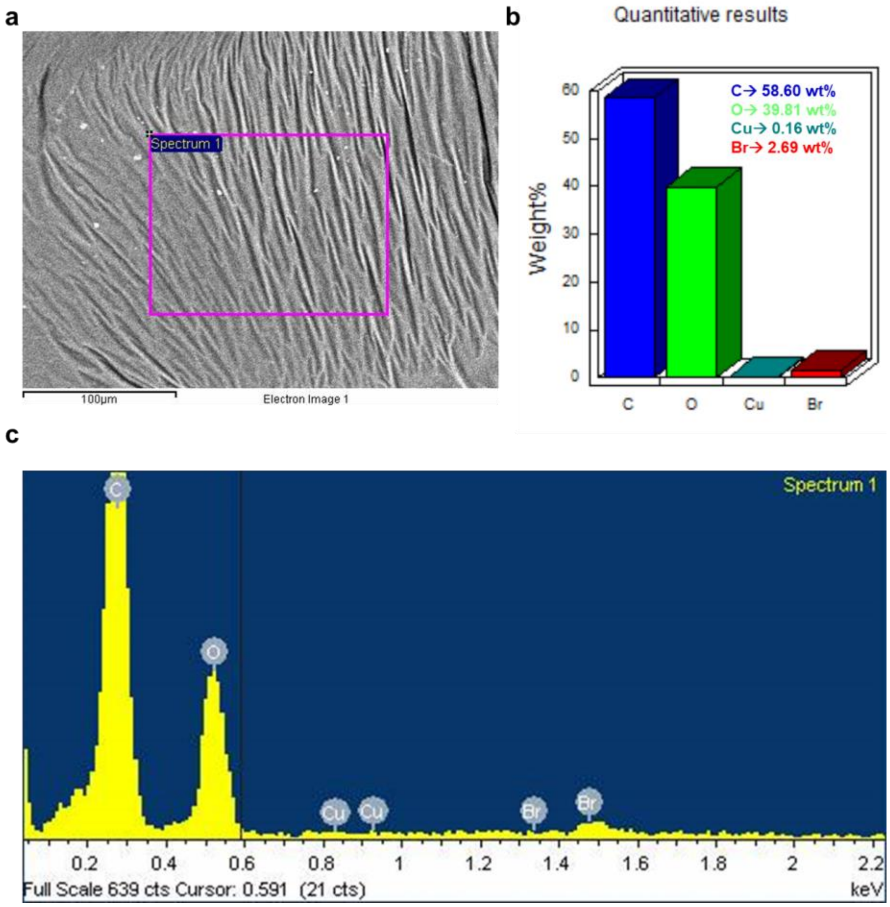

Supplementary Figure 16. EDX analysis of PBMA-GOx-chi-LNPs composite after enzyme-degassed SET-LRP-mediated Pcikering emulsion process: (a) Specific surface area used for the analysis. (b) Composition of the sample in wt% and (c) EDX spectra of PBMA-GOx-chi-LNPs using 10 kV as accelerated voltage.

Supplementary Tables

Supplementary Table 1. Nuclear magnetic resonance (NMR) spectroscopy analysis of various moieties of softwood kraft lignin used in this work by quantitative ^{13}C and ^{31}P NMR spectroscopy. Data was reproduced from a previous study.[1]

Moiety	^{31}P mmol g^{-1}	^{13}C mmol g-1	^{13}C Per 100 Ar
Total OH	6.32	6.44	107.0
Aliphatic OH	1.89	2.61	52.3
Total phenolic OH	4.09	3.56	59.2
5-free guaiacyl OH	1.94	1.70	28.3
5-substituted guaiacyl OH	1.80	1.87	31.0
p-hydroxyphenyl OH	0.31	nd	nd
OMe	-	4.37	75.3
Pinoresinol	-	0.10	1.73
Phenylcoumaran	-	0.12	2.06
β-O-4	-	0.24	4.67
β-5	-	0.12	2.06
β–β	-	0.05	0.87
G	-	2.46	41.0
H	-	0.13	2.31
Non-conjugated COOR	-	0.69	12.2
Conjugated COOR	-	0.05	0.87
Conjugated CO	-	0.22	3.83
Non-conjugated CO	-	0.37	6.31
Sugars	-	0.05	0.80

Supplementary Table 2. Characteristics of the colloidal lignin particles (LNPs) prepared in this work.[a]

Lignin form	hydrodynamic diameter (nm)	PDI	Zeta potential (mV)
LNPs[b]	97 ± 2.5	0.026 ± 0.010	-29.7 ± 3.8
chi-LNPs[c]	190 ± 2.1	0.24 ± 0.152	+31.9 ± 3.2
GOx-chi-LNPs[d]	215 ± 5.6	0.27 ± 0.096	+41.9 ± 2.0

[a]At least three measurements were completed for each parameter. Error ranges correspond to one standard deviation. [b]Values measured at native pH (3.8). [c]Values measured at pH 4 in 15 mM NaOAc buffer. [d]Values measured at pH 5.5 in 15 mM NaOAc buffer.

Supplementary Table 3. Comparison of apparent catalytic constants of free and immobilized GOx enzyme at room temperature.[a]

Enzyme status	K_m(M)	V_{max} (M s^{-1})
Free (GOx)	(5.70 ± 0.26) x 10^{-3}	2.64 x 10^{-2}
Immobilized (GOx-chi-CLPs)	(7.00 ± 0.23) x 10^{-3}	1.92 x 10^{-2}

[a]At least three measurements were completed for each parameter. Error ranges corresponds to one standard deviation.

Supplementary Table 4. Composition of oil phase and aqueous phase in the SET-LRP-mediated Pickering emulsion of BMA using GOx-chi-LNPs as stabilizers.

component[a]	weight (g)	comments
**Oil phase (organic phase)**		
BMA	1.78	20 vol % to total
MBPA	0.015	$[BMA]_0/[MBPA]_0$=200/1
Me_6-TREN	2.9 x 10^{-3}	$[BMA]_0$/[Me_6-TREN$]_0$=200/0.2
**Water phase**		
water	7.5	buffer solution pH 6.0
GOx-chi-LNPs	0.04	2.25 wt % to BMA
glucose	0.2	[glucose] = 0.14 M
Cu(0)	1.19 x 10^{-3}	$[BMA]_0/[Cu(0)]_0$=200/0.3
		Added in aqueous dispersion (0.5 g)

[a]Conditions: T = 50 ºC. V_{total} = 10 mL. The Pickering emulsion was prepared by ultrasonication and the reaction started by the addition of an aliquot (0.5 mL) of Cu(0) aqueous dispersion. See details in experimental part.

Supplementary Table 5. Average Sauter diameter and uniformity parameter of the emulsions stabilized by 2.5 wt% of GOx-chi-LNPs measured by optical microscope.

	BMA	S	MMA
Average Sauter diameter ($D_{3,2}$, μm)	28.4 ± 1.8	43.8 ± 4.5	46.7 ± 6.2
Uniformity	0.45	0.8	1.2

Supplementary Table 6. Enzyme-degassed SET-LRP-mediated Pickering emulsion of BMA for different DP_n.

entry	$[BMA]_0/[MBPA]_0$ [b]	Conv.[c] (%)	M_n[d] (th)	M_n[e]	M_w/M_n[e]
1	50/1	90	6.600	6570	1.29
2	100/1	88	12.750	15.343	1.26
3	200/1	95	27.260	29.412	1.14
4	300/1	91	39.063	42.830	1.13
5	400/1	89	50.860	53.720	1.17
6	500/1	88	62.810	65.630	1.16

[a]Polymerization conditions: BMA = 2 mL, water phase = 8 mL and $[MBPA]_0/[Me_6\text{-TREN}]_0/[Cu(0)]_0$ = 1/0.2/0.3 at 50 ºC for 12 h. [b]Calculated from the $[BMA]_0/[MBPA]_0$ ratio. [c]Determined by gravimetric analysis. [d]*M*n (th) = 142.2 x $[BMA]_0/[MBPA]_0$ x conv. + 243.1. [e]Determined by GPC in THF using PS standards.

Supplementary Table 7. Average Sauter diameter and uniformity parameter of the emulsions stabilized by 2.5 wt% of GOx-chi-LNPs measured by optical microscope.

	PBMA	PS	PMA
Average Sauter diameter ($D_{3,2}$, μm)	35.7 ± 3.8	38.2 ± 2.5	41.0 ± 1.6
Uniformity	0.56	0.43	0.32

Supplementary Methods

Materials. All chemicals were purchased from Sigma Aldrich or Fischer and used as received unless noted: Glucose oxidase (GOx) from Aspergillus niger was purchased form Sigma Aldrich as lyophilized powder and stored at -20 ºC. Peroxidase from horseradish (HDP) was purchased form Sigma Aldrich as lyophilized powder, dissolved in aqueous buffer solution (15 mM sodium phosphate, pH 6) and stored at 5 ºC. All lignin materials prepared in this work were prepared from BIOPIVA™ 100 pine kraft lignin (UPM, Finland). Buthyl methacrylate (BMA), methyl acrylate (MA), and styrene (S) were purchased from Sigma Aldrich and passed through a short basic alumina column to remove the inhibitor before the polymerization reactions.

Gel Permeation Chromatography (GPC). GPC analysis of the polymer samples was performed on a Agilent 1100 series system equipped with two gel columns (a guard column, 500 Å and PLgel 5 µm, 104 Å from Phenomenex) and a Agilent 1100 series refractive-index factor (RI) as a detector. THF (Fischer, HPLC grade) was used as an eluent at a flow rate of 1 mL min^{-1}. The calibration curves for GPC analysis were obtained with polystyrene standards purchased from PSS Polymer Standards service GmbH. The molecular weights were calculated using the universal calibration principle based on Mark–Houwink parameters.[2]

NMR Spectroscopy. ^1H NMR spectrum was recorded on a Bruker DRX400 NMR instrument at 25 °C in $CDCl_3$ containing tetramethylsilane (TMS) as an internal standard. For the chain end analysis of PMA, the delay time (D_1) was set at 10 s and the number of scans (ns) was set at 100.

Optical Microscopy. A Nikon (Alphaphot2) optical microscope was used to record the images of the emulsions before and after the polymerization. The emulsion and latex dispersions were previously diluted by adding one drop of the sample in 1 mL of deionized water. ImageJ software was used to process the recorded images and calculate the Sauter diameter ($D_{3,2}$) and uniformity (U) values based on 100 measurements for each sample.[3]

Scanning Electron Microscopy (SEM). SEM images were recorded on a JEOL JSM-7401F (JEOL Ltd., Japan) operating at 2−5 kV. Colloidal dispersions of lignin nanoparticles (LNPs, chi-LNPs and GOx-chi-LNPs) were previously diluted by a factor of 1:40, followed by the deposition and evaporation of one droplet into a silicon wafer matrix for the SEM investigation. Latex dispersions, polymeric microparticles

and cross-sections of composites, were dried on a glass substrate and sputter-coated with a thin over layer of gold to prevent sample charging effects.

Particle Size and Zeta Potential. Particle size and zeta potential of LNPs, chi-LNPs and GOx-chi-LNPs were measured using a Zetasizer Nano ZS (Malvern, UK). The zeta potential was determined using a dip cell probe. LNPs, chi-LNPs and GOx-chi-LNPs were diluted by a factor of 30 with deionized water, and sodium acetate buffer (0.1 M; pH 4 and 5.5), respectively before the analysis.

Immobilization Efficiency and Enzyme Activity. The efficiency of enzyme immobilization was assessed from protein mass balance (Bradford method, BSA standards).[1] Briefly, 100 μL of the supernatant (containing unbound GOx) was added to 40 μL of dye reagent, assuming that all unbound GOx were present in the supernatant. After 10 min, the absorbance of dye solution was measured at 595 nm. The enzyme activity and catalytic kinetic constants were determined following the formation of chromogenic hydroquinone, using benzoquinone as a substrate.[4] Briefly, the absorbance of hydroquinone (Supplementary Fig. 3a) (sodium acetate buffer, 10 mM, pH 6) was measured at 290 nm in the presence of varied concentration of D-glucose (5 mM to 100 mM) and constant amount of GOx or GOx-chi-LNPs. One unit of GOx activity is defined as the amount of enzyme that catalyzes the transformation of benzoquinone (10 mM) to hydroquinone in the mixture 250 μL at 25 ºC during 1 min. To assess thermal stability of GOx, activity measurements were made after incubation of GOx and GOx-chi-LNPs at different temperatures (20−90 ºC).

Preparation of GOx-chi-CLPs Stabilized BMA-in-Water Pickering Emulsions. All the emulsions were prepared by gradually adding BMA monomer to a water dispersion of GOx-chi-LNPs. The final fraction of oil/water was fixed at 20/80 v/v and the total volume of the emulsion was 10 mL. The final concentration of GOx-chi-CLPs was fixed to 20 g of GOx-chi-CLPs per L (2.25 wt%) of BMA. The emulsification was performed by sonication for 120 s with a BioBlock Vibra-Cell equipped with an ultrasonic tip with cooling in an ice bath (10 s on and 5 s off at 40% of amplitude power).

Preparation of block copolymers by enzyme-degassed SET-LRP-mediated Pickering emulsion process. This procedure is representative for all the chain extension experiments conducted herein. The preparation of $PBMA_{50}$-PS_{200} is described as representative example: A PBMA-Br macroinitiator was prepared as described in methods section of the main manuscript, with the following conditions: $([BMA]_0/[MBPA]_0/[Me_6\text{-TREN}]_0/[Cu(0)]_0 = 50/1/0.2/0.3$. [GOx-chi-LNPs] = 2.25 wt % relative to BMA).

After 15 h, the polymerization mixture was purified by three centrifugation/re-dispersion cycles followed by precipitation in MeOH/H_2O (8,2, v,v). The resulting PBMA-Br was dried at 50 ºC overnight (79 % yield). Stock solutions of styrene (S) (2 mL, 0.017 mmol), PBMA-Br (0.82 g, 0.082 mmol) and Me_6-TREN (4.6 μL, 0.017 mmol) were prepared. GOx-chi-LNPs (40 mg, 2.25 wt % to BMA were dispersed in a deionized aqueous solution (7.5 mL) containing glucose (200 mg, 0.14 M). Then, Pickering emulsions were prepared by ultrasonication of organic phase (oil phase) with water phase as described above. After that, the Pickering emulsion was transferred to a vial and placed in a thermostatic oil bath at 50 ºC. The introduction of an aliquot of Cu(0) powder dispersed in water (1.20 mg, 0.5 mL) started the SET-LRP process (t = 0). Reaction was allowed to proceed for 12 h and the final block copolymer was purified as described previously.

Supplementary References

[1] Sipponen, M. H., Farooq, M., Koivisto, J., Pellis, A., Seitsonen, J. & Österberg, M. Spatially confined lignin nanospheres for biocatalytic ester synthesis in aqueous media. *Nat. Commun.* **9**, 2300 (2018).

[2] Moreno, A., Bensabeh, N., Parve, J., Ronda, J. C., Cádiz, V., Galià, M., Vares, L., Lligadas, G. & Percec, V. SET-LRP of Bio- and Petroleum-Sourced Methacrylates in Aqueous Alcoholic Mixtures. *Biomacromolecules* **20**, 1816–1827 (2019).

[3] Werner, A., Sèbe, G. & Héroguez, V. A new strategy to elaborate polymer composites: Via Pickering emulsion polymerization of a wide range of monomers. *Polym. Chem.* **9**, 5043–5050 (2018).

[4] Chapman, R., Gormley, A. J., Stenzel, M. H. & Stevens, M. M. Combinatorial Low-Volume Synthesis of Well-Defined Polymers by Enzyme Degassing. *Angew. Chem. Int. Ed.* **55**, 4500–4503 (2016).
